# Assessing the Impact of Smartphone Use on Neck Pain and Related Symptoms Among Residents in Jeddah, Saudi Arabia: A Cross-Sectional Study

**DOI:** 10.7759/cureus.64299

**Published:** 2024-07-11

**Authors:** Ibrahim Hakami, Abdulhai Sherwani, Mohammed Hadadi, Riyadh Alzahrani, Abdullah Albukhari, Yazan Omar, Khalid Alsaedi, Faisal Aljadani, Najlaa Ali, Mohammed Khan, Rafal Alasmari, Amar Khan, Wasan Aleqbali, Reem Hadadi, Ghena Natto

**Affiliations:** 1 Orthopedic Surgery, Shaqra University, Shaqra, SAU; 2 Physical Therapy, Faculty of Medical Rehabilitation Science, King Abdulaziz University, Jeddah, SAU; 3 College of Medicine, Jazan University, Jazan, SAU; 4 College of Medicine, Al-Baha University, Al-Baha, SAU; 5 College of Medicine, King Abdulaziz University, Jeddah, SAU; 6 College of Medicine, Ibn Sina National College for Medical Studies, Jeddah, SAU; 7 College of Medicine, Umm Al-Qura University, Makkah, SAU; 8 College of Medicine, Fakeeh College for Medical Sciences, Jeddah, SAU

**Keywords:** saudi arabia, ergonomic practices, shoulder pain, headaches, neck pain, musculoskeletal disorders, smartphone use, text neck syndrome

## Abstract

Background: The widespread adoption of smartphones has transformed global communication but raised health concerns like Text Neck Syndrome - a musculoskeletal condition arising from prolonged device use, causing discomfort in the cervical spine. This study investigates its prevalence and associated factors among smartphone users in Jeddah, Saudi Arabia, focusing on usage patterns, symptoms, and awareness.

Methods: This cross-sectional online survey was conducted from June 1 to June 30, 2024. Data on demographics, smartphone habits, symptoms, and Text Neck Syndrome awareness were collected using a structured questionnaire. Statistical analysis involved descriptive statistics and chi-square tests.

Results: The study included 421 participants, predominantly female (279, 66.3%) and Saudi nationals (397, 94.3%). The largest age group was 21-40 years (308, 73.2%), and most were single (251, 59.6%) with a college degree (236, 56.1%). Over 42.0% of participants used smartphones for more than five hours daily, with 39.4% adopting a 30-degree neck posture. The most prevalent symptoms reported were neck pain (272, 64.6%), headaches (203, 48.2%), and shoulder pain (178, 42.3%). Awareness of Text Neck Syndrome was reported by 197 participants (46.8%), but only 60 (14.3%) had been diagnosed. Concerns about long-term complications such as osteoporosis (105, 24.9%) and prolapsed intervertebral disc (120, 28.5%) were expressed. Despite these concerns, 97.9% of participants hesitated to reduce smartphone usage due to reported symptoms.

Conclusion: This study highlights the significant musculoskeletal impact of smartphone use in Jeddah, underscoring the need for interventions promoting ergonomic practices and increasing awareness about associated risks. Public health strategies should focus on promoting ergonomic practices and educating users about preventive measures.

## Introduction

In recent years, the widespread adoption of smartphones has revolutionized communication and access to information globally [1,2]. However, this surge in usage has brought to light a range of health concerns, one of which is Text Neck Syndrome. Text Neck Syndrome refers to a musculoskeletal condition characterized by discomfort and pain in the cervical spine, resulting from prolonged and repetitive use of handheld electronic devices, such as smartphones and tablets [3,4].

The prevalence of Text Neck Syndrome has gained increasing attention as smartphones become indispensable tools in daily life, particularly among younger demographics who are heavy users [5]. The posture adopted during smartphone use is often characterized by prolonged periods of forward head flexion-places excessive strain on the cervical spine. This maladaptive posture not only affects musculoskeletal health but may also lead to a variety of associated symptoms, including neck pain, shoulder pain, upper back pain, headaches, and numbness in the hands and arms [6-8].

Research on Text Neck Syndrome has primarily focused on understanding its prevalence, associated risk factors, and potential long-term consequences. Studies have identified demographic factors, such as age and gender, as well as behavioral factors, such as duration and frequency of smartphone use, as significant contributors to the development and severity of Text Neck Syndrome symptoms [5-9].

Despite the growing recognition of Text Neck Syndrome, there remains a need for further investigation into its epidemiology, risk factors, and management strategies, particularly in diverse populations and cultural contexts. This study aims to contribute to the existing literature by examining the prevalence of Text Neck Syndrome and associated factors among smartphone users in Jeddah, Saudi Arabia. By elucidating these factors, the study seeks to inform targeted interventions and public health strategies aimed at mitigating the impact of smartphone-related musculoskeletal disorders.

## Materials and methods

Study design

This cross-sectional study aimed to investigate the prevalence of Text Neck Syndrome and associated factors among smartphone users in Jeddah, Saudi Arabia. The study was designed to collect data through an online survey conducted over one month, from June 1, 2024, to June 30, 2024. This design was chosen for its efficiency in reaching a broad and diverse participant pool within a short period, allowing for the collection of comprehensive data on smartphone usage habits, symptoms, and awareness of Text Neck Syndrome.

Study participants

Participants were recruited using convenience sampling techniques, primarily through social media platforms and online community groups to maximize reach. Eligible individuals were aged 18 years and above, residing in Jeddah, and regular users of smartphones. To ensure a representative sample, recruitment efforts targeted various demographic groups, including different age ranges, genders, and educational backgrounds. Exclusion criteria included individuals with pre-existing musculoskeletal disorders or those unable to complete the questionnaire independently due to cognitive or physical impairments.

Data collection

Data were collected using a structured questionnaire developed specifically for this study. The questionnaire was informed by existing literature on Text Neck Syndrome and smartphone usage patterns and was culturally adapted to ensure relevance to the Saudi Arabian context. It was distributed electronically via a survey platform, ensuring ease of access for participants. The questionnaire comprised several sections, including demographic information (gender, age, marital status, educational level, and nationality), smartphone usage habits (daily duration of smartphone use and habitual neck posture during use, with specific angles provided for accuracy - 0 degrees, 15 degrees, 30 degrees, 45 degrees, and 60 degrees), symptoms and diagnoses (prevalence of symptoms related to smartphone use, including neck pain, arm pain, headaches, back pain, shoulder pain, hand numbness, and stiff neck; severity of symptoms; and diagnosis of Text Neck Syndrome), awareness and attitudes (awareness of Text Neck Syndrome, perceived causes, and preventive measures, as well as attitudes towards reducing smartphone usage in light of experienced symptoms), and concerns about long-term complications (participants' concerns regarding potential long-term complications associated with prolonged smartphone use, such as osteoporosis and prolapsed intervertebral disc).

Statistical analysis

Descriptive statistics were employed to summarize the data, with frequencies and percentages used for categorical variables. Chi-square tests were conducted to explore associations between demographic factors, smartphone usage habits, and the prevalence of Text Neck Syndrome symptoms. All statistical analyses were performed using Statistical Package for the Social Sciences (IBM SPSS Statistics for Windows, IBM Corp., Version 26.0, Armonk, NY). A p-value of <0.05 was considered statistically significant.

## Results

Demographic characteristics of study participants

The study included a total of 421 participants from Jeddah, Saudi Arabia. Table 1 summarizes the demographic characteristics of the study participants. The majority were female (279, 66.3%) and Saudi nationals (397, 94.3%). The largest age group was 21-40 years (308, 73.2%), and most participants were single (251, 59.6%). Educational attainment varied, with a significant proportion having a college degree (236, 56.1%).

**Table 1:** Demographic characteristics of study participants (N = 421) Data are represented using frequencies and percentages.

Characteristics		Frequency	Percent
Gender	Male	142	33.7
	Female	279	66.3
Age Groups	≤20 years	35	8.3
	21-40 years	308	73.2
	41-60 years	73	17.3
	61+ years	5	1.2
Marital Status	Single	251	59.6
	Married	141	33.5
	Divorced	23	5.5
	Widowed	6	1.4
Education Level	Primary education	1	0.2
	Intermediate education	11	2.6
	High school diploma	142	33.7
	College degree	236	56.1
	Higher studies	31	7.4
Nationality	Saudi	397	94.3
	Non-Saudi	24	5.7

Smartphone use and neck posture habits

Table 2 presents participants' habits regarding smartphone use and neck posture. A substantial number reported using smartphones for more than five hours daily (177, 42.0%). The most common neck posture during smartphone use was at 30 degrees (166, 39.4%).

**Table 2:** Smartphone use and neck posture habits among participants (N = 421) Data are represented using frequencies and percentages.

Characteristics		Frequency	Percent
Smartphone Use Daily	Less than 2 hours	8	1.9
	2-3 hours	83	19.7
	3-5 hours	153	36.3
	More than 5 hours	177	42.0
Neck Position	0 degrees	10	2.4
	15 degrees	70	16.6
	30 degrees	166	39.4
	45 degrees	133	31.6
	60 degrees	42	10.0
Awareness of Smartphone Harms	No	35	8.3
	Yes	386	91.7

Reported symptoms and diagnoses

Table 3 outlines participants' reported symptoms and diagnoses. The most prevalent symptoms were neck pain (272, 64.6%), headaches (203, 48.2%), and shoulder pain (178, 42.3%). The severity of reported symptoms ranged from no pain (134, 31.8%) to severe pain-limiting activities (36, 8.6%).

**Table 3:** Prevalence and severity of reported symptoms (N = 421) Data are represented using frequencies and percentages.

Characteristics		Frequency	Percent
Symptoms of Smartphone Use	Experienced neck pain	272	64.6
	Experienced arm pain	163	38.7
	Experienced headache	203	48.2
	Experienced back pain	158	37.5
	Experienced shoulder pain	178	42.3
	Experienced hand numbness	139	33.0
	Experienced stiff neck	114	27.1
Text Neck Syndrome	Heard of Text Neck Syndrome	197	46.8
	Diagnosed with text neck	60	14.3
Severity of Neck Pain	No pain	134	31.8
	Mild pain with movement	89	21.1
	Mild continuous pain	130	30.9
	Severe pain	36	8.6
	Very severe pain	24	5.7
	Intolerable pain	8	1.9
Headaches Using Smartphone	No headache	157	37.3
	Mild headache	162	38.5
	Moderate headache	75	17.8
	Severe headache	22	5.2
	Persistent and severe headache	5	1.2

Awareness and attitudes towards Text Neck Syndrome

Table 4 details participants' awareness and attitudes towards Text Neck Syndrome. Nearly half of the participants (197, 46.8%) were aware of the syndrome, while a smaller proportion (60, 14.3%) reported being diagnosed with it. Multiple factors were believed to contribute to Text Neck Syndrome, including mobile device use (71, 16.9%) and e-reading devices (38, 9.0%).

**Table 4:** Awareness and attitudes towards Text Neck Syndrome (N = 421)

Characteristics		Frequency	Percent
Causes of Text Neck	Personal computer use	53	12.6
	Mobile device use	71	16.9
	E-reading devices	38	9.0
	All of the above	204	48.5
	None of the above	1	0.2
	Unsure	54	12.8
Prevention of Text Neck	No	29	6.9
	Yes	288	68.4
	Unsure	104	24.7
Symptoms of Text Neck	Chronic headaches	42	10.0
	Shoulder pain	31	7.4
	Neck pain	79	18.8
	All of the above	175	41.6
	None of the above	1	0.2
	Unsure	93	22.1

Concerns about long-term complications

Regarding participants' concerns about long-term complications associated with smartphone use, a notable percentage expressed concerns over potential complications such as osteoporosis (105, 24.9%) and prolapsed intervertebral disc (120, 28.5%). Despite the reported symptoms and potential complications, the overwhelming majority of participants (412, 97.9%) expressed hesitancy to reduce their smartphone usage.

## Discussion

This study investigated the prevalence and factors associated with Text Neck Syndrome among smartphone users in Jeddah, Saudi Arabia. The findings underscore significant insights into the musculoskeletal health implications stemming from prolonged smartphone use in this population. A notable proportion of participants reported experiencing symptoms commonly associated with Text Neck Syndrome, such as neck pain, shoulder discomfort, and headaches. These symptoms were prevalent among individuals who reported using smartphones for extended periods daily, exceeding five hours. This aligns with global concerns regarding the ergonomic impact of prolonged screen time on musculoskeletal health [10-13].

While a majority of participants demonstrated awareness of Text Neck Syndrome, understanding of preventive measures was limited. This knowledge gap underscores the importance of educational initiatives aimed at promoting ergonomic smartphone use practices to mitigate the onset of related symptoms. Specifically, interventions focusing on posture correction, regular breaks, and ergonomic accessories could potentially reduce the prevalence and severity of Text Neck Syndrome among smartphone users [10,14].

Our findings are consistent with existing research highlighting the widespread prevalence of Text Neck Syndrome symptoms among smartphone users globally. Similar studies have identified usage patterns and symptom prevalence comparable to those observed in our study population, reinforcing the global nature of this health issue [12-16].

Future research employing longitudinal designs and more diverse sampling methods could provide deeper insights into the progression and impact of Text Neck Syndrome over time. Longitudinal studies would also help in understanding how prolonged smartphone use contributes to the development of chronic musculoskeletal conditions beyond the immediate symptoms reported.

Several limitations should be considered when interpreting our findings. Firstly, the study employed convenience sampling, which may limit the generalizability of results to the broader population of smartphone users in Jeddah. The sample predominantly comprised regular smartphone users who may be more likely to experience symptoms related to Text Neck Syndrome, potentially overestimating prevalence rates compared to the general population. Secondly, the reliance on self-reported data introduces the possibility of recall bias and subjective interpretation of symptoms, despite efforts to standardize data collection through a structured questionnaire. Additionally, the cross-sectional design inherently restricts our ability to establish causal relationships between smartphone usage patterns and the development of musculoskeletal symptoms.

## Conclusions

This study provides valuable insights into the prevalence and associated factors of Text Neck Syndrome among smartphone users in Jeddah, Saudi Arabia. The findings underscore the significant burden of musculoskeletal symptoms associated with smartphone use, highlighting the need for public health interventions to promote ergonomic practices and raise awareness about the potential risks of prolonged device use. Future research should focus on longitudinal studies to elucidate the long-term implications of Text Neck Syndrome and evaluate the effectiveness of preventive measures in mitigating its impact on population health. Such efforts are crucial in addressing the growing public health concern posed by excessive smartphone usage and its associated musculoskeletal disorders.

## Appendices

**Table 5:** Survey questionnaire

Question Number	Questions	Options
Q1	Gender	Male, Female
Q2	Age Group	≤20 years, 21-40 years, 41-60 years, 61+ years
Q3	Marital Status	Single, Married, Divorced, Widowed
Q4	Educational Level	Primary education, Intermediate Education, High school diploma, College degree, Higher studies
Q5	Nationality	Saudi, Non-Saudi
Q6	How many hours do you use your smartphone daily?	Less than 2 hours, 2-3 hours, 3-5 hours, More than 5 hours
Q7	What is your habitual neck posture during smartphone use?	0 degrees, 15 degrees, 30 degrees, 45 degrees, 60 degrees
Q8	Have you experienced any of the following symptoms related to smartphone use?	Neck pain, Arm pain, Headaches, Back pain, Shoulder pain, Hand numbness, Stiff neck
Q9	Have you heard of Text Neck Syndrome?	Yes, No
Q10	Have you ever been diagnosed with Text Neck Syndrome?	Yes, No
Q11	Are you concerned about long-term complications associated with smartphone use?	Yes, No
Q12	Would you consider reducing your smartphone usage if you experience symptoms related to Text Neck Syndrome?	Yes, No
